# Ocular Manifestations of Leukemia and Results of Treatment with Intravitreal Methotrexate

**DOI:** 10.1038/s41598-020-58654-8

**Published:** 2020-02-06

**Authors:** Vicktoria Vishnevskia-Dai, Sara Sella King, Ruth Lekach, Ido Didi Fabian, Ofira Zloto

**Affiliations:** 1 Goldschleger Eye Institute, Sheba Medical Center, Tel Hashomer, Israel; 2 The Ophthalmology department, Meir Medical Center, Kfar-Saba, Israel; 3 The Sackler Faculty of Medicine, Tel Aviv University, Tel Aviv, Israel

**Keywords:** Leukaemia, Leukaemia

## Abstract

Ocular involvement in leukemia is considered rare. Ocular symptoms can be the presenting signs of leukemia, they can appear after diagnosis has been established, or they can be the first manifestation of a relapse after remission. We report, to the best of our knowledge for the first time, the ocular manifestation of a series of patients with ocular leukemia and the result of their treatment with intravitreal methotrexate (MTX) injections. This is a retrospective cohort study. The medical records of 12 consecutive patients with ocular leukemia (24 eyes, 11 eyes treated with MTX) treated at the Sheba Medical Center from January 2010 to December 2017 were retrospectively reviewed. Details on ocular inflammatory reaction and tumor cell infiltration at presentation and the end of follow-up were recorded as main outcome measures. The 12 patients included 7 women and 5 men (mean age ± standard deviation at diagnosis 25.92 ± 23.91 years, range 2–82 years). Eleven eyes of 6 patients were treated with intravitreal MTX injections. The indication for treatment was biopsy proven, tumor cell infiltration. The mean number of MTX injections was 3.37 ± 5.35 (range 1–18). The mean follow-up was 27.08 ± 36.79 months (range 1–93). All treated eyes showed improvement in the inflammatory reaction and tumor cell infiltration. In conclusion we found that Intravitreal MTX injections may be an effective therapeutic approach for eyes with intraocular leukemic tumor cell infiltration.

## Introduction

Leukemias are a group of malignant neoplasms derived from the hematopoietic stem cells as a result of abnormal proliferation of blood cells in the bone marrow^1^. Leukemia is classified according to the mode of presentation, i.e., acute or chronic, and the predominant proliferating cell type. Accordingly, the diagnosis of leukemia will be either acute myelogenous leukemia (AML), acute lymphocytic leukemia (ALL), chronic lymphocytic leukemia (CML), chronic lymphocytic leukemia (CLL), as well as other types of leukemia, such as hairy cell leukemia (HCL)^2^.

Ocular involvement in leukemia is considered rare^3,4^. Liebreich was the first to describe leukemic retinopathy in the 1860s. Ocular symptoms can present after the systemic diagnosis, they can be the presenting signs of the disease, or they can be the first manifestation of a relapse after remission^5,6^. Ophthalmic involvement is classified into 2 major categories: “primary” or direct leukemic infiltration, and “secondary” or indirect involvement. Direct leukemic infiltration can have 3 patterns:

I. Direct infiltration of the anterior segment, vitreous, choroid, and retina mimicking uveitis, choroiditis and retinitis.
II. Infiltration of the optic nerve presenting with or without another cranial nerve involvement (cranial nerves III, IV, or VI) clinically mimicking palsies and swollen discs.
III. Infiltration of the orbit mimicking orbital inflammatory diseases. Intraocular leukemic involvement most frequently presents in the posterior segment. Hemorrhages in the retina are the most prevalent feature of leukemia^4,7^. Some forms of hemorrhages representing leukemic cell infiltration while others forms of hemorrhages in leukemic patients are secondary to the systemic conditions. Secondary hemorrhages can present as dots or blots, flame-shaped, etc. Additionally, the hemorrhage may extend into the subretinal or vitreous spaces. Cotton wool spots in leukemic patients may be attributed to nerve fiber layer infracts or to localized collections of leukemic cells. Peripheral microaneurysms and peripheral neovascularization are also ocular signs of chronic leukemia^8,9^.

The current treatment of patients with ocular leukemia includes systemic chemotherapy and biological treatments^10,11^. In some cases, however, it may not be adequate since penetration of systemic chemotherapy to the involved ocular structure may be insufficient. Another treatment option is irradiation, but it frequently involves local complications. Therefore, treatments with intravitreal injections of dexamethasone^12^, anti–vascular endothelial growth factor (anti-VEGF)^13^, or methotrexate (MTX)^14^ have been recently investigated.

The purpose of this study is to summarize our experience with the ocular manifestations and outcomes of patients with ocular leukemia and to present, for what we believe to be the first time, a series of patients with ocular leukemia treated with intravitreal MTX injections.

## Methods

### Patients

The medical records of all consecutive patients that presented with ocular leukemia and who were treated at the Ocular Oncology Service at the Goldscleger Eye Institute, Sheba Medical Center from January 2010 to December 2017 were retrospectively reviewed. The diagnosis of leukemia was based on the patient’s medical history, systemic clinical features, complete blood count and differential results, findings of a bone marrow aspiration and a hematologic evaluation. On completion of the hematologic evaluation, patients with ocular complaints underwent a comprehensive ophthalmic examination and anterior or vitreous tapping for cytology. The follow-up of these patients continued until there has been clinical or imaging evidence of improvement of their ocular status, or systemic disease progression that precluded the relevance of ocular findings, or demise.

### Data collection

The following data was retrieved for all patients with ocular leukemia: demographics, medical history, laterality of the ocular condition, ocular presentation, treatment method, treatment result, and both the ocular and systemic disease outcome. The ocular examination was performed with Snellen VA converted to log minimum angle of resolution (MAR) values at the beginning and end of follow-up.

This case series was approved by the local institutional review board (IRB) of Sheba Medical Center and informed consent for study participation was taken. All methods were performed in accordance with to National Institutes of Health guidelines

### Intraocular biopsy

Intraocular biopsy was done on anterior chamber tap (when cells were detected in the anterior chamber) or vitreous biopsy.

Intravitreal biopsy was done in any patient with involvement of the posterior segment in order to rule out leukemia or exclude other reasons that may cause posterior segment infiltration in immunosuppression patients such as infectious etiologies.

The biopsy was performed with 30-gauge needle aspiration from the anterior chamber or suture less combined 25-gauge vitrectomy. The specimens (0.1 ml of anterior chamber fluid or 0.3–0.5 ml of clear vitreous) was fixated and prepared as cell block paraffin and stained with hematoxylin and eosin, Gram and periodic acid-Schiff stains. Immunohistochemical staining for CD3, CD20 and CD79a were performed. Moreover, PCR tests for bacteria and viruses were performed.

None of the patients underwent therapeutic vitrectomy.

### MTX treatment

The indication for treatment was biopsy proven, tumor cell infiltration and not any other secondary form of intraocular involvement, such as anemia, hyperviscosity and thrombocytopenia.

The relevant patients were treated with repeated intravitreal MTX 400 mg/0.1 ml, twice weekly in the first month, once weekly in the following two months and once in a month thereafter. The treatment regimen was based on the known beneficial treatment response of patients with vitreoretinal lymphoma to intravitreal MTX injections^15^.

The indication for treatment completion was clinical resolution of the inflammatory response along with regression of tumor cell infiltrate (vitreous, optic nerve, macula, and retina) or patient systemic deterioration and inability to attend our clinic.

### Statistical analysis

Quantitative variables were described as mean, range, and standard deviation. Categorical variables were described as absolute and relative frequencies. Chi-square analysis were used to calculate proportional difference between presentation to the end of follow-up. The overall significance level was set to an alpha of 0.05. The statistical analysis was carried out using Microsoft Excel 2017 (Microsoft Corporation, Redmond, WA) and IBM SPSS software version 24.0 (SPSS, Inc., Chicago, IL, USA).

## Results

### Demographics

Between January 2010 and December 2017, a total of 24 eyes of 12 patients (5 men and 7 women, mean age 25.92 ± 23.91 years, range 2–82 years) were diagnosed with ocular leukemia.

### Leukemia types

Seven patients had ALL, 3 patients had Acute promyelocytic leukemia, one patient had AML, and one patient had HCL. CNS involvement was present in 5 (41.65%) patients and absent in the other 7 patients (58.33%).

### Ocular manifestation

In three patients (25%) the first manifestation of the leukemia was ocular, 4 patients (33.3%) had known leukemia at the time of the ocular involvement diagnosis and 5 patients (41.65%) the first manifestation of disease relapse was ocular.

The most common reason for referral to ophthalmic examination was a decrease in visual acuity (n = 9, 75%) followed by ocular pain (n = 2, 16.66%). One patient (8.33%), a 2-year-old child, was diagnosed during a routine eye examination. The ocular manifestations at presentation are summarized in Table 1.

**Table 1:** Ocular Manifestations of Leukemia.

Ocular involved part	At Presentation, N	At the End of Follow-up, N	*P* Value
**Anterior chamber**			0.024*
No involvement	18 (75%)	24 (100%)	
Cells	3 (12.5%)	0 (0%)	
Hyphemia	3 (12.5%)	0 (0%)	
**Vitreous**			0.210
No involvement	17 (70.83%)	19 (79.16%)	
Cells	3 (12.5%)	3 (12.5%)	
VH	2 (8.33%)	2 (8.33%)	
**Retina**			0.043*
No involvement	13 (54.16%)	17 (70.83%)	
Hemorrhage	4 (16.67%)	2 (8.33%)	
Malignant cells	2 (8.33%)	0 (0%)	
Serous RD	2 (8.33%)	0 (0%)	
**Macula**			0.366
No involvement	13 (54.1%)	4 (16.67%)	
ME	0 (0%)	1 (4.16%)	
Scar	0 (0%)	4 (16.67%)	
Infiltrates	2 (8.33%)	0 (0%)	
Hemorrhages	1 (4.16%)	1 (4.16%)	
Exudates	4 (16.67%)	0 (0%)	
Microaneurysm	1 (4.16%)	0 (0%)	
CWS	2 (8.33%)	1 (4.16%)	
Hemorrhages with white			
Infiltrates	2 (8.33%)	1 (4.16%)	
**Optic nerve**			0.163
No involvement	16 (66.67%)	14 (68.33%)	
Direct malignant cell infiltrates	8 (33.32%)	1 (4.16%)	
Pale disk	0 (0%)	6 (25%)	

^*^Significant (paired sample test). VH = vitreous hemorrhage; RD = retinal detachment; ME = macular edema; CWS = cotton wool spot.

During the follow-up period, one patient developed CMV retinitis and one patient developed graft-versus-host disease which presented mainly as severe keratopathy.

### Treatment

The ocular and systemic treatment methods are summarized in Table 2.

**Table 2:** Ocular and Systemic Treatment Methods.

Treatment	N (%)
**Ocular treatment, eyes**	
None	12 (50)
MTX 400 µg/0.1 ml intravitreal injection	6 (25)
Bevacizumab 1.25 mg/0.5 ml intravitreal injection	4 (16.67)
Ganciclovir 2 mg/0/1 ml intravitreal injection	1 (4.16)
MTX + bevacizumab intravitreal injections	1 (4.16)
**Systemic treatment, patients**	
None	0 (0)
Allogenic BMT	1 (8.33)
TBI + BMT	5 (41.67)
Brain radiation	4 (3.33)
Intrathecal MTX	3 (25)
Chemotherapy	12 (100)

MTX = methotrexate; BMT = bone marrow transplantation; TBI = total body irradiation.

### Patients treated with intravitreal MTX

Eleven eyes of 6 patients (3 men, 3 women) were treated with repeated intravitreal MTX injections according to the above protocol. All treated eyes had a positive biopsy. Six other patients didn’t have anterior chamber cells or infiltrative disease involving the posterior segment so they didn’t undergo anterior chamber tap or vitreal biopsy.

The mean age of the patients that were injected, was 15.50 ± 10.59 years (range 1–85).

**Figure 1:**
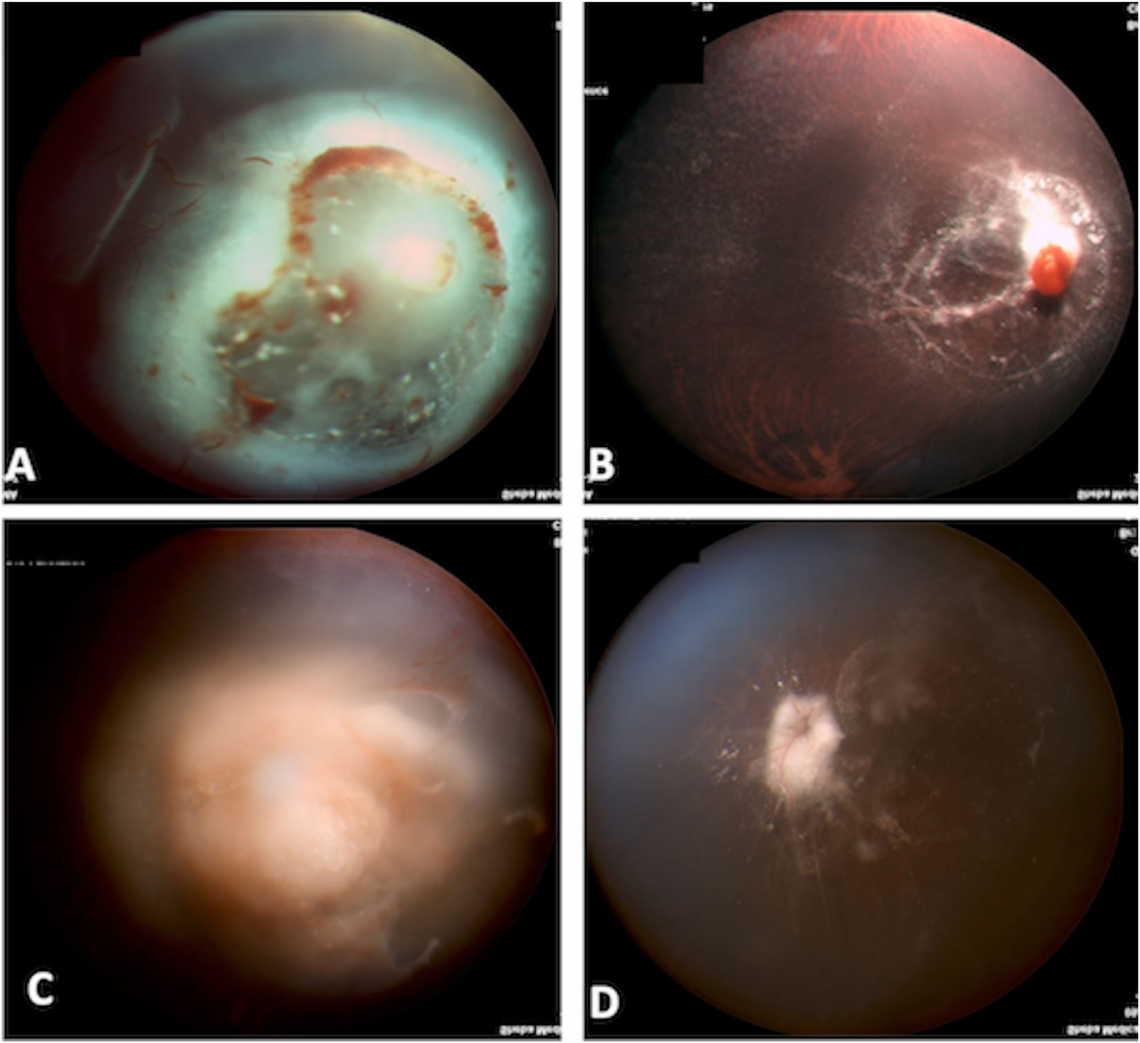
A 3-year-old child diagnosed as having leukemia presented with posterior segment and optic nerve involvement. (**A**) Right eye, before treatment: massive infiltration of the optic nerve and macula with extensive serous retinal detachment involving most of the retina and an attached retinal only in the superior part. (**B**) Right eye, after treatment: absorption of the massive infiltrate and resolution of the retinal detachment. (**C**) Left eye, before treatment: massive infiltration of the optic nerve and vitreous cavity. (**D**) Left eye, after treatment: absorption of the massive infiltration of the optic disc and vitreous cavity.

**Figure 2:**
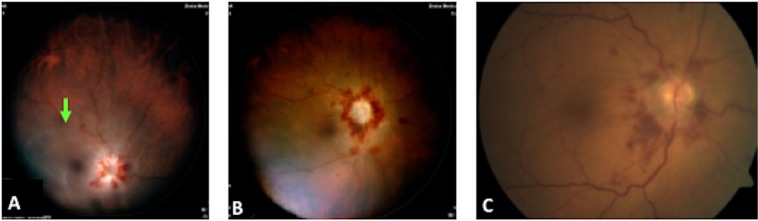
A 56-year-old female diagnosed as having precursor B cell acute lymphocytic leukemia who presented with blurred vision and black spots on her right eye. (**A**) Right eye, before treatment: clusters of cells in the vitreous cavity (arrow), swollen disc with white massive infiltration, and flame-shaped hemorrhage. (**B**) After a series of 10 intravitreal methotrexate (MTX) injections 400  mg/0.1 ml demonstrating clearance of the vitreous cells and full absorption of the optic nerve infiltration. (**C**) After a series of 14 intravitreal MTX injections.

The mean number of MTX injections was 3.37 ± 5.35 (range 1–18). Reasons for treatment completion were - clinical resolution of the inflammatory response with regression of tumor cell infiltrate in 4 patients (a mean of 5.25 ± 2.23 injections, rang: 5–18 injections) (Figs. 1, 2). Systemic deterioration that precluded the relevance of ocular findings, or demise in 2 patients (a mean of injections 1.50 ± 0.50 injections, rang: 1–3 injections). Those 2 patients didn’t show significant improvement. However, we speculate that if they could continue the injections additional improvement may have been seen.

The ocular involvement in 5 of the patients who received intravitreal MTX developed following the presentation of the systemic disease, while it was the first sign of a relapse in one patient.

The mean VA of the treated group was 1.20 Log MAR at presentation and 1.30 Log MAR at the last follow-up (*P* = 0.255, matched pair analysis). Four eyes presented with an anterior chamber (AC) tumor cell infiltration and inflammatory reaction, which resolved after treatment with intravitreal MTX in all eyes (*P* = 0.05, match pair analysis).

**Figure 3:**
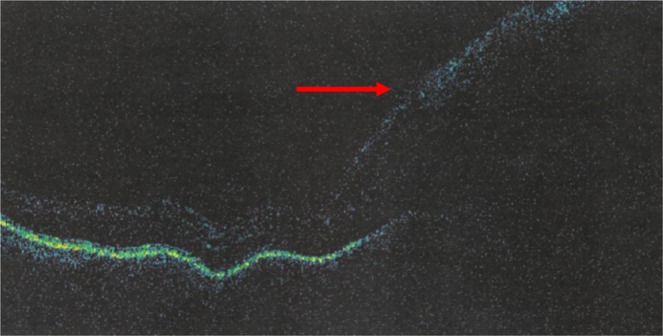
Right eye optical coherence tomography time domain featuring the infiltrate around the optic disc.

**Figure 4:**
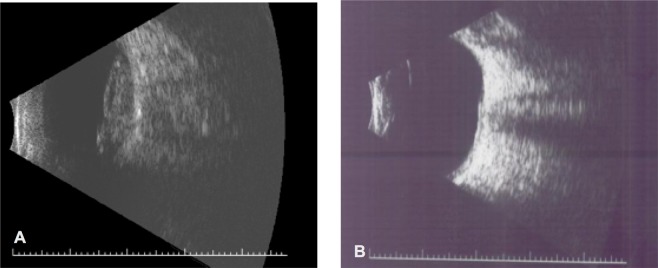
Ultrasound of right eye with massive sub retinal leukemia infiltration and exudation. (**A**) Before injections. (**B**) After 8 injections.

Eight eyes presented with vitreitis and swollen disc secondary to direct tumor cell infiltration (Figs. 3, 4), all of which had resolved at the end of follow-up, although 4 patients had pale discs. Table 3 summarizes the findings at presentation and at the end of follow-up in the intravitreal MTX-treated group.

**Table 3:** Ocular Presentation in Patients Treated with Methotrexate (n = 11 eyes).

Manifestation	Before Treatment N (%)	After Treatment N (%)	*P* Value
**Anterior chamber**			
Cells	4 (36.36)	0 (0)	0.032*
**Vitreous**			
Vitritis	8 (72.72)	0 (0)	0.022*
**Retina**			0.114
No involvement	6 (54.54)	5 (45.45)	
Hemorrhages	0 (0)	2 (18.18)	
Exudates	2 (18.18)	2 (18.18)	
Microaneurysm	2 (18.18)	0 (0)	
**Macula**			0.751
No involvement	4 (36.36)	4 (36.36)	
Scar	2 (18.18)	2 (18.18)	
Infiltrates	2 (18.18)	0 (0%)	
Hemorrhages	0 (0)	2 (18.18%)	
Exudates	0 (0)	1 (9.09%)	
Microaneurysm	1 (9.09)	0 (0%)	
**Optic nerve**			0.291
No involvement	7 (63.63)	7 (63.63)	
Direct malignant cell infiltrate	4 (36.36)	0 (0)	
Pale disk	0 (0)	4 (36.36)	

There was no difference between the 6 patients (11 eyes) in the intravitreal MTX-treated group and the rest of the patients at the end of follow-up with regard to the findings at the AC, vitreous, macula, and retina (*P* = 0.990, 0.327, 0.417, and 0.213, respectively).

With the exception of mild corneal epitheliopathy in one patient, there were no complications as a result of the intravitreal MTX injections in this group of patients.

### Survival outcomes

The mean duration of follow-up was 27.08 ± 36.79 months (range 1–93). One patient completed only follow up of one month because of a rapid systemic deterioration and death. At the end of follow-up, 7 patients (58.3%) were alive: 6 patients were in disease remission and one patient whose ocular leukemia had resolved was under maintenance treatment of low-dose chemotherapy for the systemic disease. The ocular manifestations at the end of follow-up vs. at presentation are summarized in Table 1.

## Discussion

Ocular symptoms can be the presenting signs of leukemia, they can appear after diagnosis has been established, or they can be the first manifestation of a relapse after remission. This study summarized the presentation, treatment, treatment results, and survival outcomes of 12 patients with ocular leukemia. Eleven patients were referred to ophthalmic evaluation because of decreased VA. The ocular findings in the first examination included: tumor cell infiltration and inflammatory reaction in the AC, tumor cell infiltrates and hemorrhages in the retina, choroid, vitreous and/or optic disc. All of those findings resolved at the end of follow-up. Eleven eyes were treated with intravitreal injections of MTX, after which the inflammatory reaction was cleared and tumor cell infiltration resolved.

Patient referral for ophthalmic examination was based on patient complaints (pain or a decrease in VA that can be explained by the clinical findings at presentation). However, incidental diagnosis of ophthalmic involvement also encountered during a routine ocular examination in one of our youngest patients.

Notably, ALL is the single most common malignancy affecting children who usually cannot express their complaints^16^. We speculate that under diagnosis of ophthalmic involvement may be prevalent in this group of leukemia patients. Therefore, we believe that all patients with leukemia in all age groups should be screened with at least one ophthalmic examination at presentation and then undergo routine examination every 6 months (or earlier if there are ocular complaints).

Traditionally, patients with leukemia and ocular manifestations are treated systemically with anti–leukemia-targeted methods or irradiation. Leukemic patients who presented with choroidal neovascularization had been treated effectively with intravitreal anti-VEGF injections^13,17^. Treatment with reparative intravitreal MTX injections, for vitreoretinal lymphoma, has been reported and accepted as an efficacious therapeutic option during the past 20 years^15^. However, although the proliferative cancer cell is of the same origin, intravitreal MTX injections for treating intraocular leukemia was reported only once in a single case report from 2018 by Mello *et al*.^14^.

In this current study, we described a series of 11 eyes with intraocular leukemia that were treated with intravitreal MTX injections. Following the treatment, the VA did not change, yet reduced inflammatory reaction in the AC, resolution of swollen disc, and absorption of AC, retinal and disc tumor cell infiltrates was achieved. No resolution of retinal hemorrhages was detected in any of our patients who were treated with MTX. Retinal hemorrhages may be attributed to indirect leukemic hematologic changes, such as low blood cell counts, rather than direct tumor cell infiltration. In addition, at the end of follow-up, patients that were treated with MTX had more pale discs that can be related to the regression of the swollen disc caused by direct tumor cell infiltration. No patient in this study developed side effects from the injections apart from mild transient corneal epitheliopathy in one patient.

Based on our findings and results we suggest that repetitive intravitreal MTX injections, may be considered as an adjunctive treatment method combined with intrathecal treatment when no systemic active disease is present in the blood and bone marrow. The limitations of this study resulted from its retrospective nature, the small number of eyes in our series and the fact that systemic disease progression in some cases preclude the relevance of ocular findings. A larger, randomized controlled trial is required to examine the effectiveness of this treatment and related side effects in comparison to systemic chemotherapy and irradiation. Moreover, further larger studies that compare between patients with infiltrative disease with positive biopsy treated with IVT MTX vs. patients with positive biopsy not treated with IVT MTX and patients with infiltrative disease with negative biopsy, should be done in order to isolate the treatments additive value

In conclusion, we presented a series of 12 patients with ocular leukemia, of whom 6 (11 eyes) were treated with intravitreal MTX injections that led to improvement in their inflammatory reaction and tumor cell infiltration. To the best of our knowledge, this is the first case series to describe this treatment approach for intraocular leukemia.
